# Quinupristin/dalfopristin in Staphylococcus aureus endophthalmitis: a case report

**DOI:** 10.1186/1752-1947-5-130

**Published:** 2011-03-31

**Authors:** Sergio E Hernandez-Da Mota

**Affiliations:** 1 Blvd. García de León 598. Colonia Nueva Chapultepec CP, 58280, Morelia, Michoacán, México

## Abstract

**Introduction:**

The intravitreal injection of antibiotics remains the mainstay of therapy for postoperative endophthalmitis. Bacterial resistance, however, is still a pitfall in achieving an adequate response to treatment. Quinupristin/dalfopristin might be a feasible therapeutic option in these cases.

**Case presentation:**

A 55-year-old Hispanic man had endophthalmitis secondary to *Staphylococcus aureus *in his right eye and was treated with intravitreal 0.4 mg/0.1 ml quinupristin/dalfopristin injection. Inflammation and pain remission were observed at four days after injection. The final best-corrected visual acuity was 20/40.

**Conclusion:**

Although vancomycin remains the first-line intravitreal antibiotic therapy against infectious endophthalmitis caused by Gram-positive bacteria, quinupristin/dalfopristin exhibits similar efficacy and is theoretically more active against vancomycin-resistant strains, with no apparent retinal toxicity.

## Introduction

Postoperative endophthalmitis has been a major and dreaded complication in all types of eye surgery. Several measures have been used for its prevention, such as aseptic techniques and topical prophylactic antibiotics. Although these measures have resulted in very low endophthalmitis incidence [1], they have not been able to eliminate it completely.

Once infection is identified, the mainstay of therapy has been the intravitreal injection of antibiotics [2]. The concomitant use of pars plana vitrectomy and steroids has also been recommended [3] in some cases.

The most common bacterial etiology of the disease involves Gram-positive bacteria, especially *Staphylococci*. Vancomycin, a glucopeptide antibiotic, has shown adequate activity against this type of bacteria [4].

Nonetheless, as with all kinds of antibiotics, resistance that could preclude a favorable outcome in these patients may emerge [5].

Quinupristin/dalfopristin (Q/D), a recently developed streptogramin antibiotic, has shown better activity against vancomycin-resistant strains of staphylococci [6].

The purpose of this report is to describe a case in which intravitreal quinupristin/dalfopristin injection in endophthalmitis due to *Staphylococcus *sp. resulted in remission of the infection.

## Case presentation

A 55-year-old Hispanic man, in whom non-complicated phacoemulsification surgery had been performed in the right eye, exhibited a sudden decrease in visual acuity, intense pain, and marked red eye two days later.

**Figure 1:**
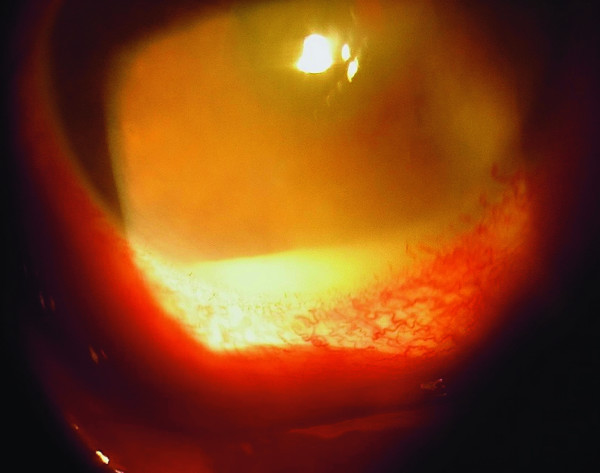
**Endophthalmitis OD showing marked ciliary reaction, hypopion, and corneal edema**.

The visual acuity was hand movement, with an intense ciliary reaction, hypopion, and moderate corneal edema (Figure 1). A diagnosis of endophthalmitis was then made.

B-scan echography showed increased intravitreal echoes due to the severe inflammatory reaction. A pars plana vitrectomy was performed, and a vitreous biopsy specimen was obtained. A combination of quinupristin/dalfopristin (0.4 mg/0.1 ml; Synercid; DSM Pharmaceuticals, Inc., Greenville, NC), ceftazidime (2.25 mg/0.1 ml) (Fortum; GlaxoSmithKline, Mexico DF, Mexico), and dexamethasone (0.4 mg/0.1 ml) (Alin, Chinoin, Mexico DF, Mexico) was injected intravitreally.

A coagulase-negative *Staphylococcus aureus *strain that was vancomycin resistant was identified through culture analysis.

**Figure 2:**
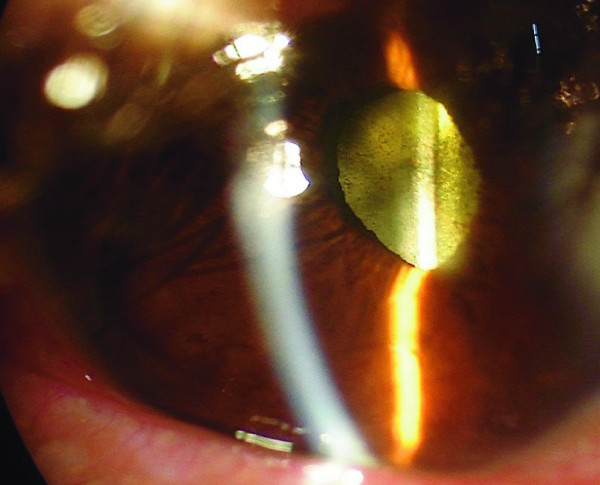
**Hypopion and inflammation remission 4 days after intravitreal antibiotic injection**.

Four days after intravitreal injection, total remission of pain and hypopion was observed (Figure 2). Vitreous opacities had cleared at the two-month follow-up. The final best-corrected visual acuity was 20/40.

## Discussion

Infectious endophthalmitis treatment has evolved with time. The condition was a common complication in the pre-Listerian era and the cause of many disastrous outcomes in intraocular surgery.

Now, although uncommon, endophthalmitis remains one of the most feared complications of intraocular surgery [1]. The gold standard of treatment has been the intravitreal administration [2] of antibiotics over topical, subconjunctival, or systemic routes.

Several modifications have been made to the combination of antibiotics given intravitreally before the causative agent is known. These modifications are mainly based on retinal toxicity and bacterial susceptibility to different antibiotics. The combination of antibiotics that is currently preferred empirically includes vancomycin and ceftazidime.

Vancomycin is a glucopeptide antibiotic that is active against Gram-positive bacteria [4]. Ceftazidime, a third-generation cephalosporine, is active against Gram-negative bacteria, especially enterobacteria.

Although vancomycin has not been overused and is reserved as a third-line antibiotic against serious infections (for example, sepsis), certain staphylococci strains have developed resistance [5]. This could be a potential source of therapeutic failure in some cases of endophthalmitis.

Q/D is a combination of two streptogramins: quinupristin (a B streptogramin) and dalfopristin (an A streptogramin) at a 30:70 ratio, respectively. The combination acts as a rapid bactericide, except for its action against *Enterococcus faecium*, which is less. The combination has a prolonged antibiotic effect (up to 10 hours) for *Staphylococcus aureus *compared with other antibiotics. Its spectrum of coverage includes Gram-positive cocci, including strains that are resistant to other antibiotics. Methicillin-sensitive and resistant strains of *Staphylococcus aureus *are also covered by Q/D.

The minimal inhibitory concentrations are 1 mg/ml or less. To the best of our knowledge, no reports of ocular toxicity exist [6]. Given this excellent anti-staphylococcal profile, the absence of ocular toxicity reports, the prolonged antibacterial effects, and the fact that no vancomycin was available at the time, we decided to treat the patient with the antibiotic combination described. Q/D, conversely, is a more recent antibiotic, which, theoretically, is less likely to induce the development of resistant strains than is vancomycin. The pharmacokinetics and pharmacodynamics of intravitreal Q/D have not yet been studied.

Our patient exhibited an adequate response to the use of intravitreal Q/D that was documented four days after injection. This response is similar to that shown to vancomycin. Notably, the use of the combination carries with it the potential advantage that we could be fighting strains that are resistant to vancomycin. Moreover, a synergistic effect of the vancomycin-quinupristin/dalfopristin combination has been documented against some Gram-positive bacteria [7]. We believe that the favorable outcome in our patient was achieved, in order of decreasing importance, by the following factors: 1) Q/D; 2) vitrectomy, which removed infected vitreous strands; and 3) dexamethasone, which limited the severity of inflammation. Ceftazidime affects mainly Gram-negative bacteria, so it probably did not play an important role in controlling the infection process.

Another issue that should be addressed is the fact that Q/D is an expensive medication, and its cost may outweigh its benefit. Moreover, the intravitreal route of this antibiotic is off-label for infectious endophthalmitis.

## Conclusion

In conclusion, Q/D showed a positive outcome in this case of infectious postoperative endophthalmitis caused by Gram-positive bacteria. To the best of our knowledge, this is the first report on the use of intravitreal quinupristin/dalfopristin in infectious Staphylococcus endophthalmitis. This report does not recommend Q/D as a first-line choice for conventional treatment of infectious endophthalmitis. Rather, we describe a positive outcome in one case.

## Patient's perspective

I write the following to provide assistance to the case report written about my operation. I have no medical knowledge or background, so I only write from my own perspective and experience. I arrived at the hospital with severe eye pain and completely blurred vision in my right eye a few days after my cataract surgery. I then was told that a very bad infection had taken over my eye. I was told that one of the specific antibiotics for this type of infection was not available, but that a non-approved one could be of benefit, so I agreed to be treated with the non-approved antibiotic via eye injection, in combination with other drugs as well as a vitrectomy surgery, which was performed subsequently. A few hours later, the pain slowly decreased until I did not feel it any more. My sight was still blurred for about a month and a half, but it slowly came back. I still see some little dots, but my vision seems okay now.

## Abbreviations

Q/D: quinupristin/dalfopristin.

## Consent

Written informed consent was obtained from the patient for the treatment and the publication of this case report and any accompanying images. A copy of the written consent is available for review by the Editor-in-Chief of the journal.

## Competing interests

The author declares that they have no competing interests.

## Authors' contributions

The author was in charge of the patient's treatment and follow-up, as well as the echography study. He was also responsible for the manuscript drafting and revision.
